# *Lilium pumilum* stress-responsive NAC transcription factor *LpNAC17* enhances salt stress tolerance in tobacco

**DOI:** 10.3389/fpls.2022.993841

**Published:** 2022-09-02

**Authors:** Yiping Wang, Ying Cui, Bin Liu, Ying Wang, Shaoying Sun, Jingwen Wang, Mengmeng Tan, Hao Yan, Yanni Zhang

**Affiliations:** College of Landscape Architecture, Northeast Forestry University, Harbin, China

**Keywords:** *Lilium pumilum*, LpNAC17, salt stress, transcription factor, ornamental plant

## Abstract

*Lilium pumilum* is a perennial herb with ornamental edible and medicinal value. It is an excellent wild germplasm resource with wide distribution and strong resistance. The NAC family of transcription factors is unique to higher plants. The NAC family plays a regulatory role in plant growth and development and participates in plant responses to biotic and abiotic stresses. The *LpNAC17* gene of *L. pumilum* was cloned and transformed into tobacco to investigate the response of transgenic tobacco to salt stress. The results showed that the net photosynthetic rate and contents of chlorophyll in *LpNAC17* over-expressed tobacco were higher than those in the control plants, while the stomatal conductance, transpiration rate and intercellular CO_2_ concentration were lower than those in the controls. The activity of superoxide dismutase, peroxidase, catalase, and the content of proline in *LpNAC17* over-expressed tobacco were higher than those in the controls, while the content of malondialdehyde, superoxide anion, and hydrogen peroxide were lower than that in the control. Nitro-blue tetrazolium staining and 3,3′-diaminobenzidine tissue localization showed that the contents of ${\text{O}}_{2}^{-}$ and H_2_O_2_ in transgenic tobacco was lower than in the controls. The expression levels of *NtSOD, NtPOD, NtCAT, NtHAK1, NtPMA4*, and *NtSOS1* in the transgenic tobacco were higher than those in the controls. Therefore, this study provides a gene source for molecular breeding of salt-tolerant plants through genetic engineering, and lays a foundation for further research on salt-tolerant Lily.

## Introduction

Plants are often affected by various abiotic stresses including the stresses of salt, drought, extreme temperature (high temperature, low temperature) during their growth and development. These stresses seriously affect the ecological and economic value of plants (Zhang and Ma, 2019). Thus, plants have to respond to these stresses to minimize the stress-induced damage (Pérez et al., 2013). Many studies have shown that plant cells can sense stresses and transmit stress signals to stress-responding transcription factors (TFs) through complex signaling pathways. TFs can regulate the expression of target genes in plants by binding its DNA binding domain to *cis*-acting elements in the promoter regions of target genes (Karam et al., 2002; Chen and Zhu, 2004; Huang et al., 2012). Many TF families such as the NAC, bZIP, MYB, and WRKY families have been found to play important roles in plant growth and development, and responses to abiotic stresses (Dortje et al., 2011). The *NAC* gene family is a large family of transcription factors unique to plants. So far, there are 117 *NAC* genes in *Arabidopsis thaliana*, 151 *NACs* in rice (Ooka et al., 2003; Mohammed et al., 2010), 163 *NACs* in *Populus trichocarpa* (Rui et al., 2010), 152 *NACs* in soybean (Dung et al., 2011), 283 *NACs* in upland cotton (Sun et al., 2018), 152 *NACs* in maize (Kaliyugam et al., 2014), 154 *NACs* in tobacco (Li et al., 2018), 168 *NACs* in durum wheat (Saidi et al., 2017). NAC TFs participate in plant morphogenesis, root growth and elongation, and the responses to various abiotic stresses. In *Rosa chinensis* “Old Blush,” the expression of *RcNAC72* was significantly induced by drought, cold, salinity, and abscisic acid (ABA), and overexpression of *RcNAC72* in *A. thaliana* enhanced its tolerance to drought stress (Jia et al., 2022). Knockout of *OsNAC3* reduced rice sensitivity to ABA and increased its sensitivity to salt stress, whereas overexpression of *OsNAC3* had the opposite effect (Zhang et al., 2021). Similarly, silencing and overexpression of the *SlNAC6* gene in tomato decreased and enhanced tolerance to drought, respectively (Jian et al., 2021). *MdNAC047* in apple (*Malus domestica*) enhances tolerance to salt stress by inducing ethylene accumulation (An et al., 2018). In the analysis of the expression pattern of *SmNAC*s in eggplant, it was found that they are involved in the regulation of responses to a variety of abiotic stresses (Wan et al., 2021). In addition, the Pearl millet (*Pennisetum glaucum*) *PgNAC21*, the horsegram (*Macrotyloma uniflorum*) *MuNAC4*, and the tomato (*Solanum lycopersicum*) NAC transcription factor JUNGBRUNNEN1 (*JUB1*) also positively regulate responses to abiotic stresses (Pandurangaiah et al., 2014; Thirumalaikumar et al., 2018; Harshraj et al., 2019). However, studies have shown that NAC transcription factors also exhibit a negative regulatory role in plant responses to abiotic stresses, e.g., *ANAC069* increased sensitivity to salt and osmotic stress by reducing reactive oxygen species scavenging capacity (He et al., 2017). Similarly, *ZmNAC071* enhanced the sensitivity to ABA and osmotic stress by downregulating the expression of superoxide dismutase (*SOD*), peroxidase (*POD*), and other genes in transgenic *A. thaliana* (He et al., 2019). Therefore, NAC transcription factors regulate stress tolerance in plants through multiple pathways and are important for plants to cope with abiotic stress.

*Lilium pumilum* is a perennial wild herb. Its flower shape is beautiful and has high ornamental value. Moreover, its bulb and flower have edible and medicinal values. It is mainly distributed in Northeast and Northern China. It has a strong tolerance to cold, drought, and saline-alkali stresses and a strong resistance to diseases. It is an excellent wild germplasm resource (Wang et al., 2018a,b). So far, a small number of genes have been cloned from *L. pumilum* and transferred into tobacco. Studies have shown that over-expression of *L. pumilum LpNAC13* and *LpPEX7* genes can enhance salt and alkali tolerance in tobacco (He et al., 2020; Wang et al., 2020). The salt tolerance of *L. pumilum APX* and *LpNAC6* overexpression plants was significantly enhanced under salt stress (Chen et al., 2010; Liu et al., 2020). However, there are still many valuable NAC transcription factors that have not been functionally characterized in *L. pumilum*. In the present study, we found that *LpNAC17* in *L. pumilum* responds to salt stress according to the analysis of transcriptome data and *NAC* gene expression. We cloned the *LpNAC17* gene from *L. pumilum* and transformed it into tobacco. We found that the overexpression of the *LpNAC17* gene in tobacco significantly improved the salt resistance of transgenic plants, showing enhanced photosynthetic rate, osmotic regulation substance content, and antioxidant enzyme activities of transgenic tobacco under salt stress. These results indicated that *LpNAC17* was positively correlated with salt tolerance, permitting further research on the molecular mechanism of *LpNAC17*-mediated regulation of salt stress in *L. pumilum*.

## Results

### Identification of the *LpNAC17* gene in *L. pumilum*

**Figure 1:**
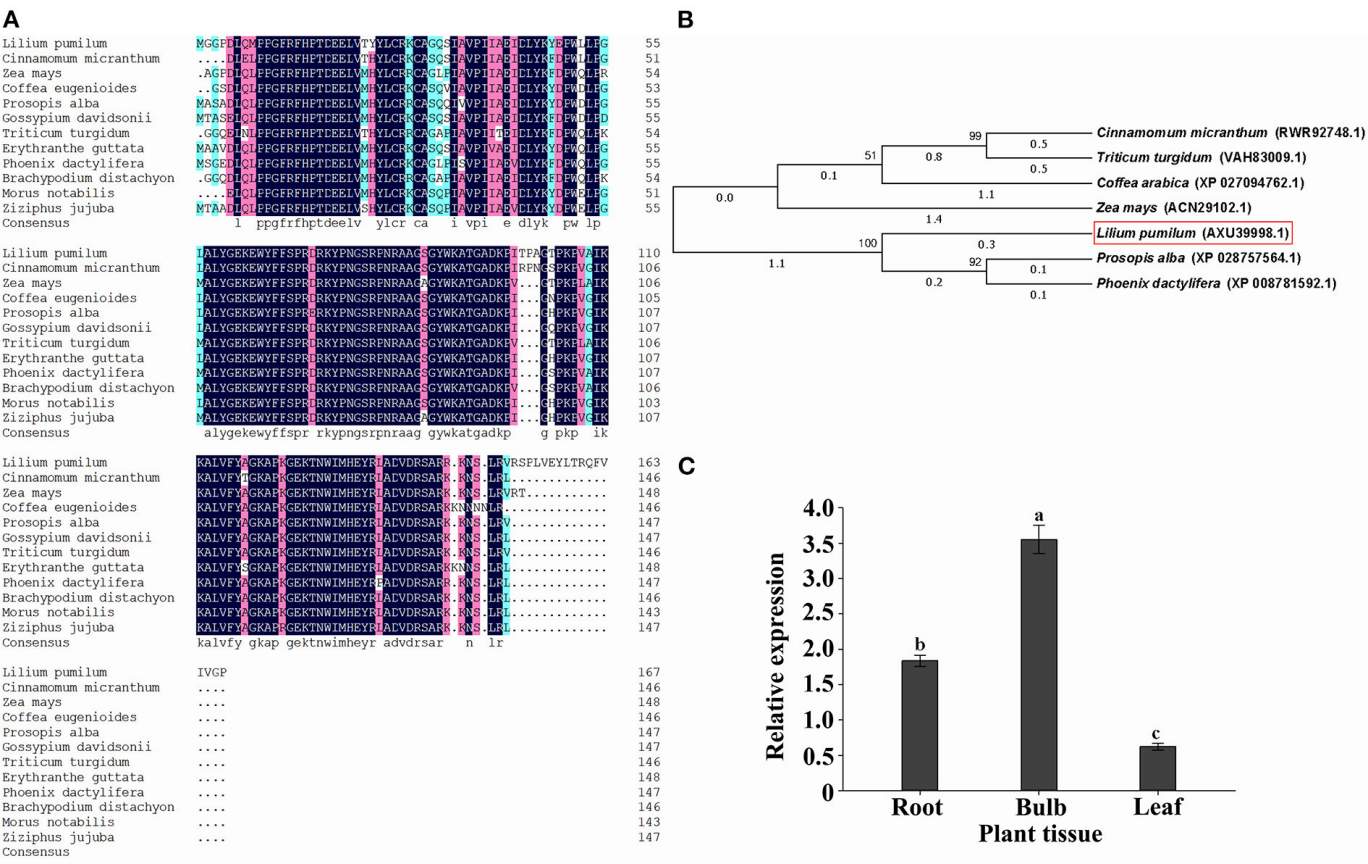
Sequence and expression analysis of *LpNAC17*. **(A)** Multiple sequence alignment of the deduced amino acid sequences of *LpNAC17* from *L. pumilum* with other NACs from *Cinnamomum micranthum* (RWR92746.1), *Zea mays* (ACN29102.1), *Coffea eugenioides* (XP_027148352.1), *Prosopis alba* (XP_028757564.1), *Gossypium davidsonii* (MBA0625903.1), *Triticum turgidum* (VAH67189.1), *Erythranthe guttata* (XP_012844117.1), *Phoenix dactylifera* (XP_008781592.1), *Brachypodium distachyon* (XP_003564785.1), *Morus notabilis* (XP_010095416.1), *Ziziphus jujuba* (XP_015885739.1). **(B)** Phylogenetic tree of LpNAC17 and its homologs. **(C)** Expression analysis of *LpNAC17* gene in different tissues of *L. pumilum*. LilyActin was used as the internal control gene. Bars represent the SE of three independent experiments and the different letters indicate the significant difference at *P* < 0.05.

Sequence analysis indicated that the open reading frame (ORF) of *LpNAC17* gene is 507 bp long and encodes a NAC gene family protein of 168 amino acids in length. The molecular weight of LpNAC17 was 19021.02 kDa, and the theoretical isoelectric point was 9.64, indicating that LpNAC17 was a hydrophilic stable protein. The results of the prediction of the transmembrane structure and signal peptide showed that the protein does not contain a transmembrane domain or signal peptide, and is a non-secreted protein. A Blast search of Genbank showed that the highest sequence homology between *L. pumilum* and *Cinnamomum micranthum* was 91.1% for *LpNAC17* (Figure 1A), and the phylogenetic analysis showed that *LpNAC17* forms a monophylogenic group with its homologs in *Prosopis alba* and *Phoenix dactylifera* (Figure 1B). Real-time RT-PCR (qPCR) analysis showed that *LpNAC17* was expressed in the root, bulb, and leaf of *L. pumilum* with the highest expression level in bulb (Figure 1C).

### Expression of the *LpNAC17* gene in *L. pumilum* under abiotic stress

**Figure 2:**
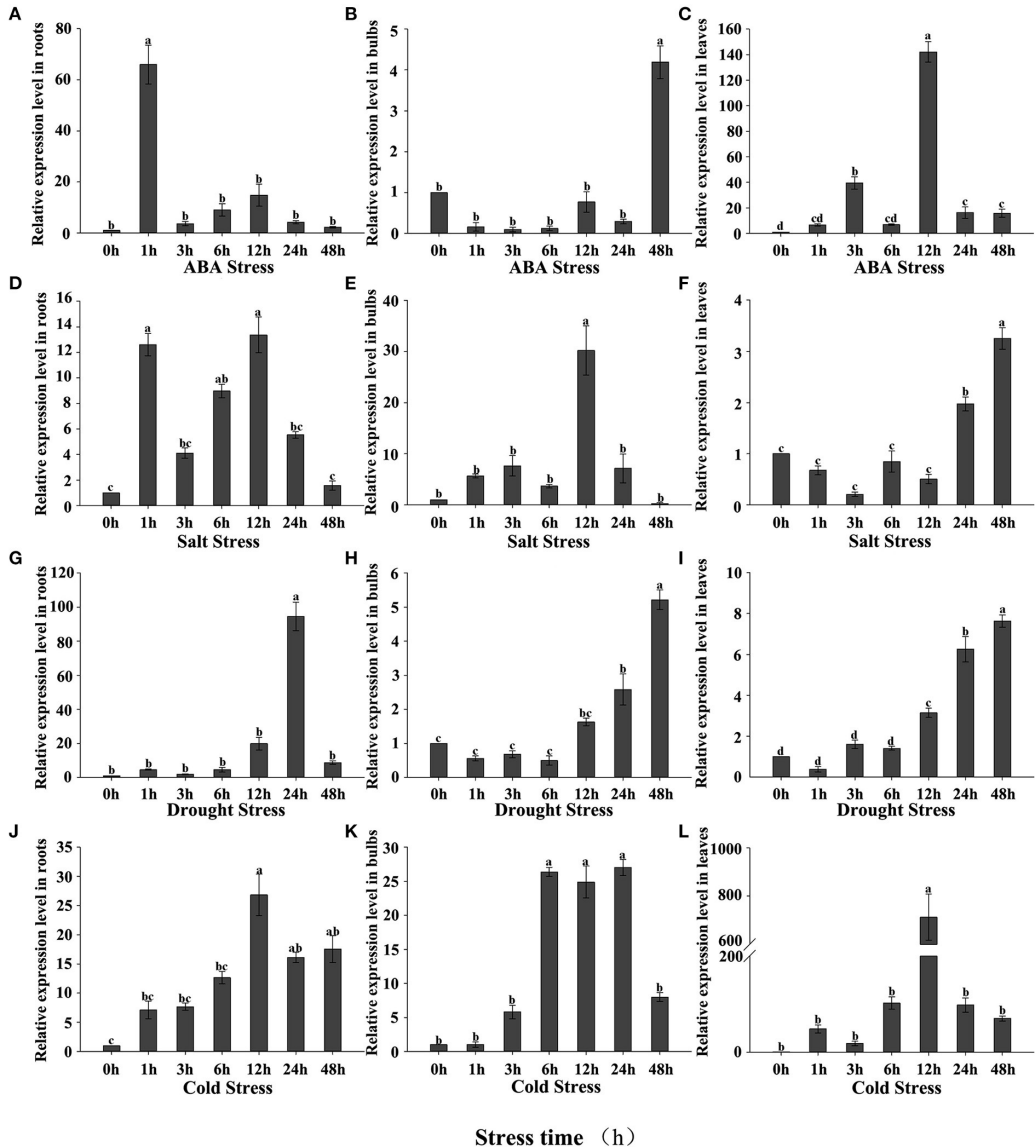
Expression patterns of *LpNAC17* in roots, bulbs, and leaves of *L. pumilum* under abiotic stress treatment. **(A–C)** 150 μM ABA treatment. **(D–F)** 200 mM NaCl treatment. **(G–I)** 20% PEG-6000 treatment. **(J–L)** 2°C treatment. Bars represent the SE of three independent experiments and the different letters indicate the significant difference at *P* < 0.05.

We investigated how the expression of *LpNAC17* in *L. pumilum* seedlings responded to the individual treatments of ABA, NaCl, drought and cold at 1, 3, 6, 12, 24, 48 h post treatment (hpt). Under ABA treatment, the expression levels of *LpNAC17* in roots, bulbs and leaves were induced when compared with that in the mock-treated plants within 48 hpt (Figures 2A–C). The highest induced expression was 58.08 times in roots at 1 hpt, 3.97 times in bulbs at 48 h, and 141.04 times in leaves at 12 h, respectively. Under NaCl stress, the *LpNAC17* expression in the three tissues were increased when compared with that in the mock-treated plants within 48 hpt (Figures 2D–F). The highest induced expression was 12.12 times in roots at 12 hpt, 31.41 times in bulbs at 12 hpt, and 3.24 times in leaves at 48 hpt. For the drought stress, the *LpNAC17* expression in the three tissues were also induced when compared with that in the mock-treated plants within 48 hpt (Figures 2G–I). The highest induced expression was 86.82 times in roots at 24 hpt, 5.18 times in bulbs at 48 hpt, and 7.46 times in leaves at 48 hpt. The cold stress treatment also induced the *LpNAC17* expression in the three tissues within 48 hpt (Figures 2J–L). The highest induced expression was 12.46 times in roots at 12 hpt, 26 times in bulbs at 24 hpt, and 714.58 times in leaves at 12 hpt. As a result, the expression levels of *LpNAC17* in the three tissues were significantly induced by each of the ABA, NaCl, drought, and cold treatments.

### Generation of transgenic tobacco lines overexpressing *LpNAC17*

**Figure 3:**
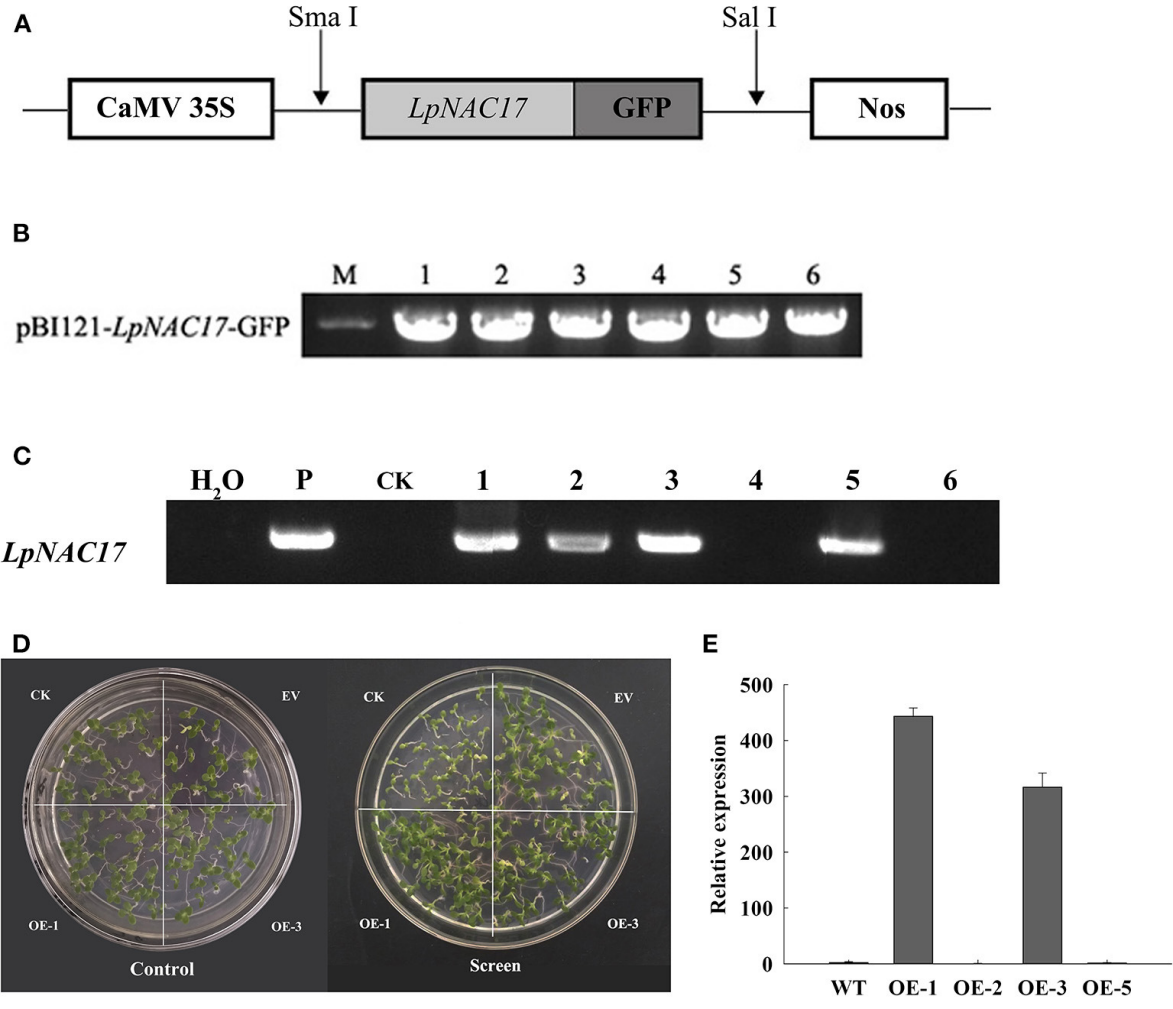
Generation of transgenic tobacco overexpressing *LpNAC17*. **(A)** Schematic diagram of T-DNA region of the expression vector containing *35S: LpNAC17-NosT*. **(B)** Construction of binary vector pBI121- *LpNAC17*-GFP. M, 2000 DNA marker; 1–6, verification of binary vectors. **(C)** PCR confirmation of the presence of *LpNAC17* in transgenic tobacco lines. P, plasmid DNA as a positive control; H_2_O, sterile distilled water as a negative control; CK, wild-type tobacco; #1, 2, 3, and 5, transgenic tobacco lines. **(D)** Growth of CK, EV and the OE-1 and OE-3 lines on the media containing Kanamycin. **(E)** Relative expression of *LpNAC17* in the transgenic tobacco lines.

The ORF of *LpNAC17* was cloned into the pBI121 vector to form pBI121-*LpNAC17*- GFP (Figures 3A,B), followed by *Agrobacterium*-mediated tobacco transformation. A total of 6 potential transgenic lines with normal growth and development were obtained. PCR amplification of the transgene *LpNAC17* confirmed the presence of the transgene in 4 out of the 6 potential transgenic lines (Figure 3C). qPCR analysis of the relative expression levels of *LpNAC17* in the four transgenic lines showed that the single-copied homozygous OE-1 and OE-3 lines had high expression levels (Figure 3E), whose seedlings grew normally in the Kanamycin selection media (Figure 3D). Thus, these two lines were selected for subsequent experiments.

### The effect of the overexpressed *LpNAC17* on salt tolerance

**Figure 4:**
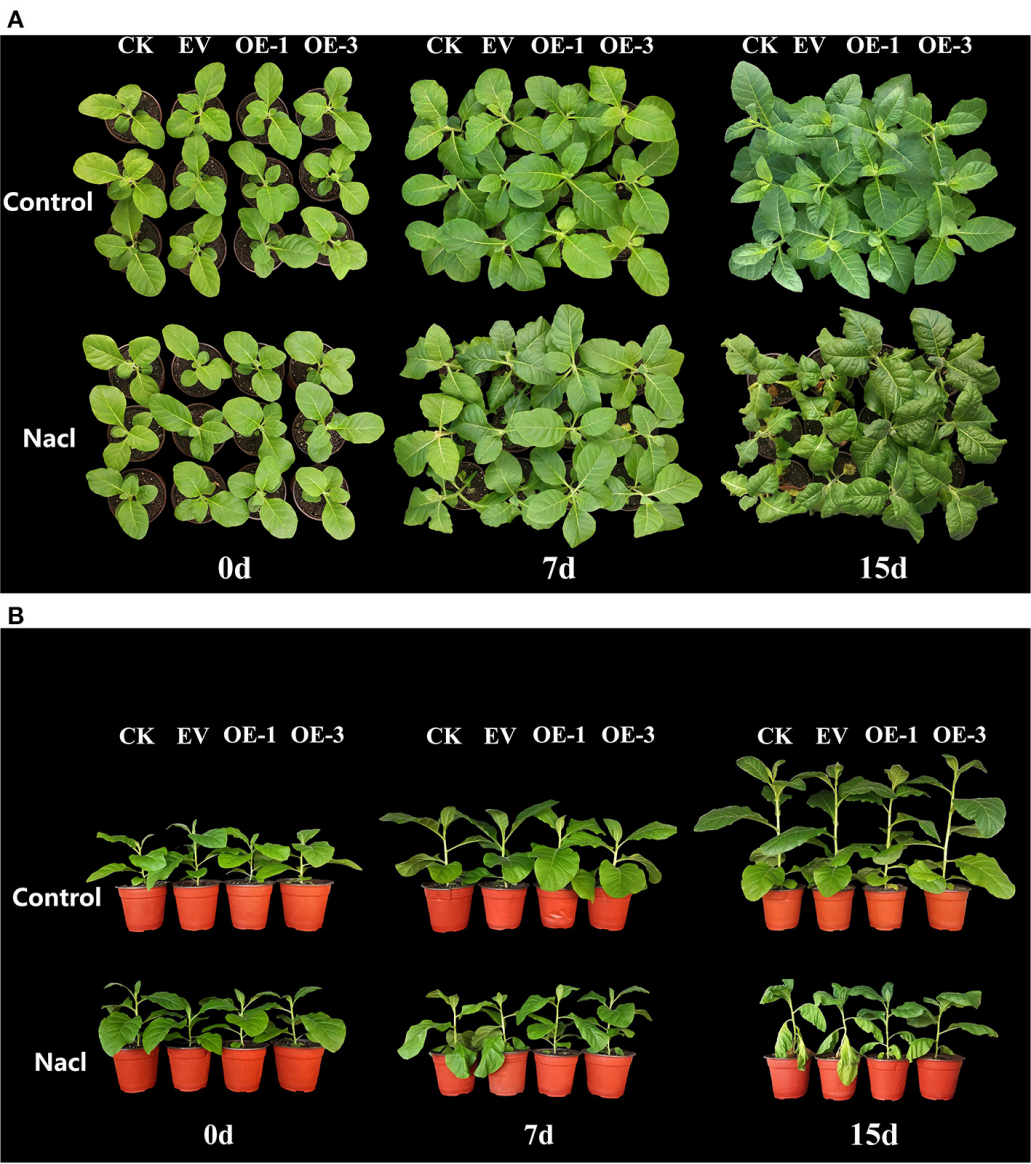
Analysis of salt tolerance in the transgenic tobacco lines overexpressing *LpNAC17*. **(A)** Comparative top view images. **(B)** Comparative side view images.

To explore whether the transgenic tobacco lines overexpressing *LpNAC17* are tolerant of salt stress, the 5-week-old wild-type tobacco (CK), transgenic tobacco lines with the empty vector (EV) and the OE-1 and OE-3 lines were used for salt stress treatment. There was no apparent difference in the morphology of the tobacco plants at 0 day post treatment (dpt; Figures 4A,B). After 7 dpt, 2–3 leaves on the lower part of each of CK and EV tobacco plants began to wilt, but the growth of OE-1 and OE-3 plants was not affected. After the 15 dpt, the leaves on the lower part of the CK and EV tobacco plants started to turn yellow and drooped, and the upper leaves began to wilt, while the leaves near the root of OE-1 and OE-3 lines just began to wilt, indicating an enhanced tolerance to salt stress in the transgenic tobacco lines overexpressing *LpNAC17*.

### The effect of the overexpressed *LpNAC17* on photosynthetic capacity under salt stress

**Figure 5:**
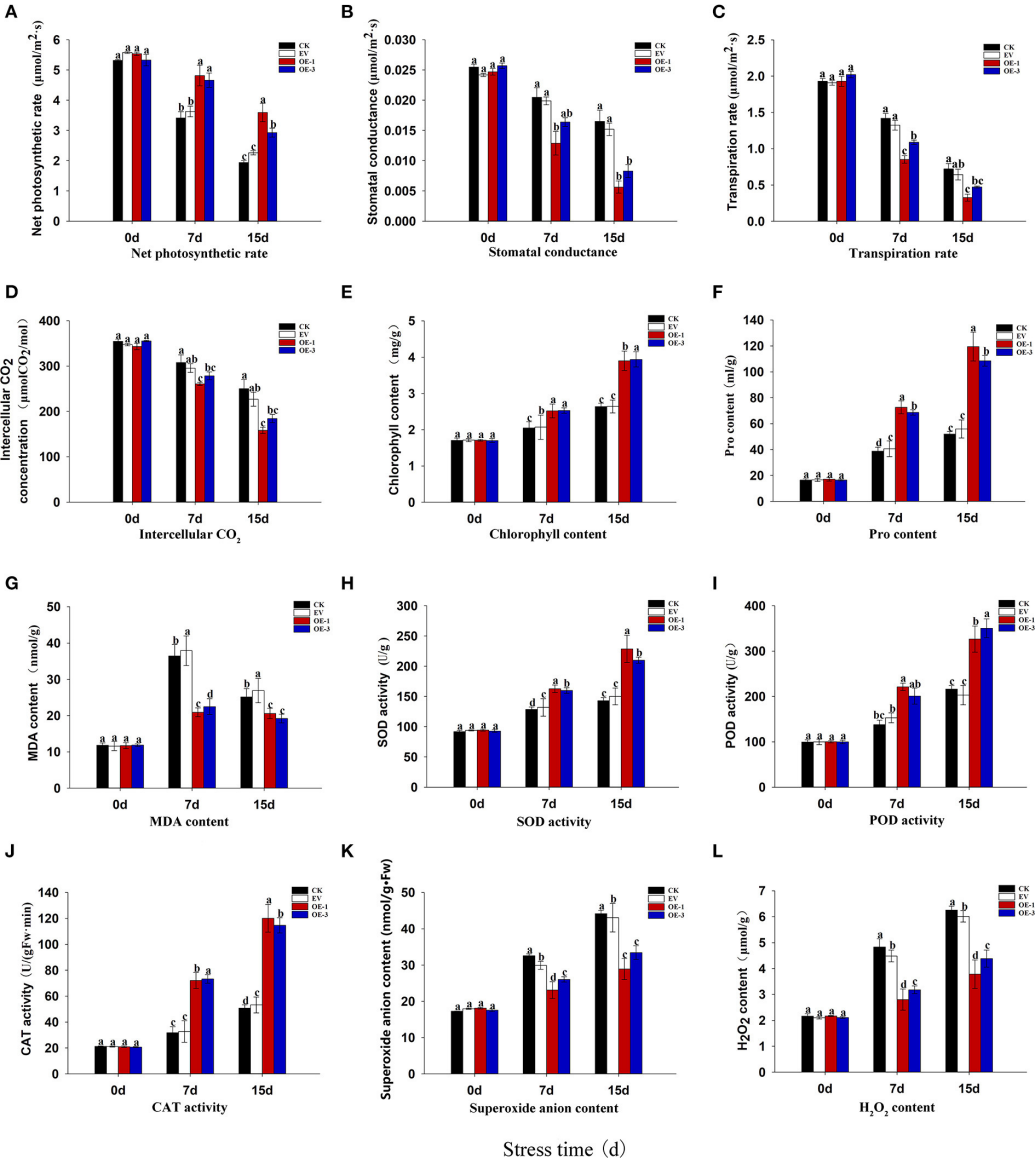
Analysis of photosynthetic capacity in the transgenic tobacco lines overexpressing *LpNAC17* under salt stress. **(A)** Net photosynthetic rate (Pn). **(B)** Stomatal conductance (Gs). **(C)** Transpiration rate (Tr). **(D)** Intercellular CO_2_ concentration (Ci). **(E)** Chlorophyll (Chl) content. **(F)** Proline (Pro) content. **(G)** malondialdehyde (MDA) content. **(H)** Superoxide dismutase (SOD) activities. **(I)** Peroxidase (POD) activities. **(J)** catalase (CAT) activities. **(K)** Superoxide anion (${\text{O}}_{2}^{-}$) content. **(L)** Hydrogen peroxide (H_2_O_2_) content. Bars represent the SE of three independent replicates and the different letters indicate the significant difference at the same time point (*P* < 0.05).

Photosynthesis is the basis of energy conversion and the most sensitive physiological activity to salt stress in plants. Net photosynthetic rate (Pn) can directly reflect the photosynthetic capacity of plants, since it is positively correlated with the latter. We found that the Pn of all of the tobacco leaves decreased gradually with the increase in salt stress treatment duration (Figure 5A). However, the Pn of OE-1 and OE-3 lines was significantly higher than that of CK and EV at 7 and 15 dpt (*P* < 0.05), and the Pn of OE-1 and OE-3 lines was 1.85 and 1.29 times higher than that of CK at 15 dpt.

The stomatal conductance (Gs), transpiration rate (Tr), and intercellular CO_2_ concentration (Ci) of all the tobacco plants showed an obvious decreasing trend with the increase in the stress treatment duration (Figures 5B–D). However, the decrease degrees of these three parameters in the OE-1 and OE-3 lines were significantly greater than that of CK and EV (*P* < 0.05). The Gs, Tr, Ci of the OE-1 and OE-3 lines were decreased by 65.79 and 49.67%, 54.80 and 34.52%, 36.75 and 26.36%, respectively, when compared with CK at 15 dpt, indicating that salt stress had significant inhibitory effect on the three parameters, and the inhibitory effect on the OE-1 and OE-3 lines was more obvious than in the CK and EV. The decrease in Ci may be caused by the joint action of Gs and Pn. The decrease in Gs restricts the entry of external gas into tobacco leaves, while the increase in Pn consumes more carbon dioxide in leaves, thus leading to the decline in Ci in tobacco leaves.

Chlorophyll (Chl) synthesis is an important component in plant photosynthesis. At 15 dpt, the Chl content of all the tobacco leaves increased continuously, and the OE-1 and OE-3 lines showed a significantly higher Chl content than that of CK and EV (Figure 5E). The Chl contents in the OE-1 and OE-3 lines were 1.47 and 1.49 times higher than that of CK, respectively, at 15 dpt. The increase of Chl content may be one of the reasons for the increased Pn in tobacco.

### The effect of the overexpressed *LpNAC17* on ROS levels and ROS scavenger activities under salt stress

The proline (Pro) contents were gradually increased in all the tobacco plants with the increase in salt stress treatment time, while the Pro contents in the OE-1 and OE-3 lines were significantly higher than that of CK and EV at 7 and 15 dpt (Figure 5F). The malondialdehyde (MDA) contents in all the tobacco plants increased at 7 dpt and then decreased at 15 dpt (Figure 5G). However, the MDA content in the OE-1 and OE-3 lines was significantly lower than that in CK and EV at both time points.

To study the effect of the overexpressed *LpNAC17* on reactive oxygen species (ROS) homeostasis, we examined the activities of key ROS scavengers superoxide dismutase (SOD), peroxidase (POD), and catalase (CAT). There was no significant difference between treatments at 0 dpt. The activities of the SOD (Figure 5H), POD (Figure 5I), and CAT (Figure 5J) in all the tobacco leaves were gradually increased when plants were subjected to salt stress, and their activities in the OE-1 and OE-3 lines were significantly higher than those of CK and EV at 7 and 15 dpt (*P* < 0.05).

**Figure 6:**
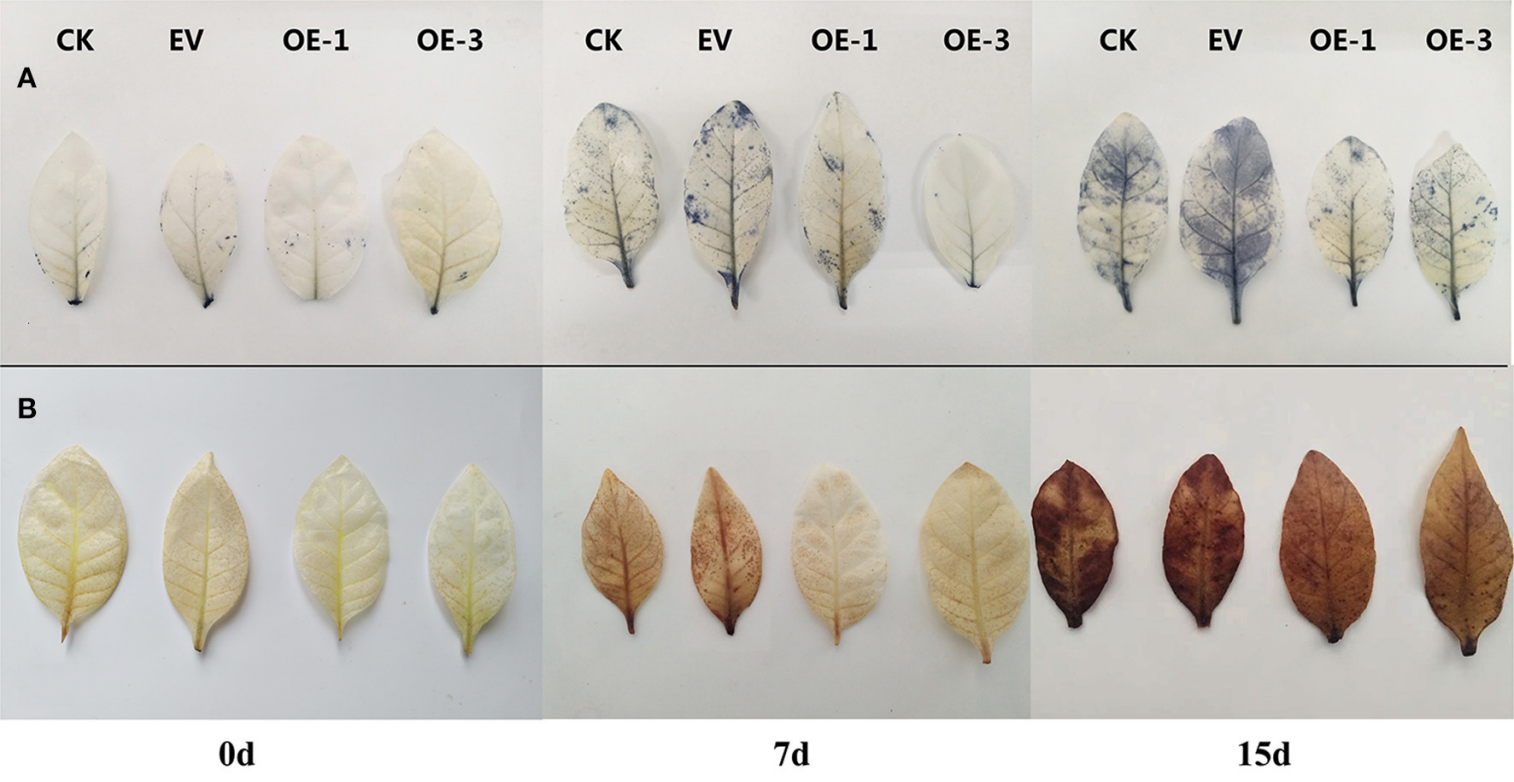
Histochemical staining of the leaves of the transgenic tobacco lines overexpressing *LpNAC17* under salt stress. Accumulation of **(A)** O_2_^−^and **(B)** H_2_O_2_ was revealed by histochemical staining with NBT and DAB, respectively.

In order to further determine the ROS content in tobacco leaves, we measured the contents of superoxide anion (${\text{O}}_{2}^{-}$) and hydrogen peroxide (H_2_O_2_). The contents of ${\text{O}}_{2}^{-}$ and H_2_O_2_ gradually accumulated in all the tobacco plants with the increase in salt stress treatment time (Figures 5K,L). However, the contents of ${\text{O}}_{2}^{-}$ and H_2_O_2_ in the OE-1 and OE-3 lines were significantly lower than that in CK and EV at 7 and 15 dpt (*P* < 0.05). Histochemical staining analysis using nitro-blue tetrazolium (NBT; for ${\text{O}}_{2}^{-}$) and 3,3′-diaminobenzidine (DAB; for H_2_O_2_) staining showed the leaves of the OE-1 and OE-3 lines were stained much lighter than that of the CK and EV at 7 and 15 dpt (Figures 6A,B), indicating less contents of ${\text{O}}_{2}^{-}$ and H_2_O_2_ in the OE-1 and OE-3 lines. These results were consistent with the content determination of ${\text{O}}_{2}^{-}$ and H_2_O_2_ in Figures 5K,L.

### The effect of the overexpressed *LpNAC17* on expression levels of stress-related genes *NtSOD, NtPOD, NtCAT, NtHAK1, NtPMA4*, and *NtSOS1* under salt stress

**Figure 7:**
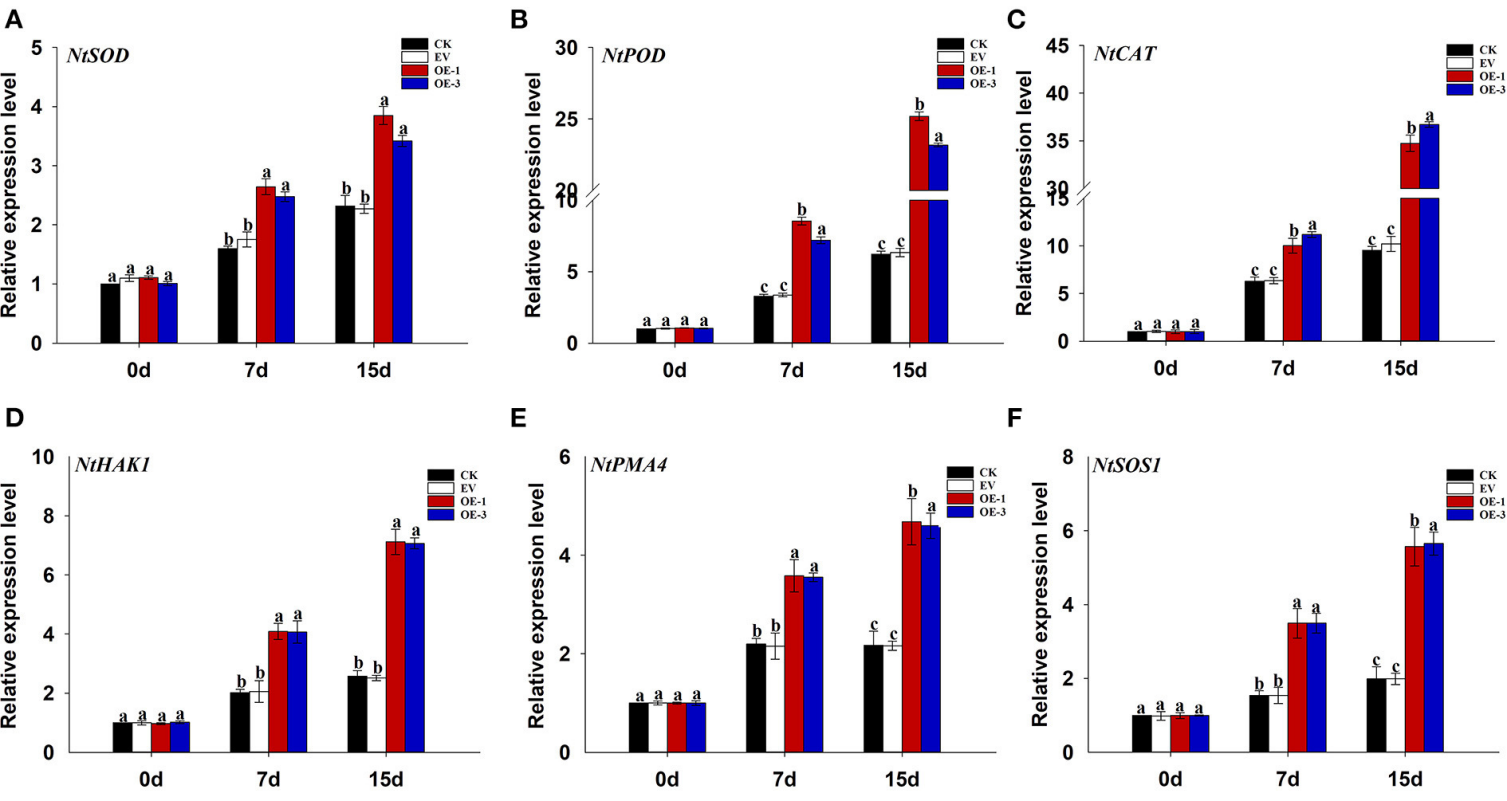
Analysis of the changes in the expression of stress-related genes in the leaves of the transgenic tobacco lines overexpressing *LpNAC17* under salt stress. **(A)** *NtSOD*. **(B)** *NtPOD*. **(C)** *NtCAT*. **(D)** *NtHAK1*. **(E)** *NtPMA4*. **(F)** *NtSOS1*. The tobacco *Actin* gene was used as the internal control gene. Bars represent the SE of three independent replicates and the different letters indicate the significant difference at the same time point (*P* < 0.05).

In order to further verify the role of *LpNAC17* in salt stress tolerance, the relative expression levels of stress-related genes *NtSOD, NtPOD, NtCAT, NtHAK1, NtPMA4*, and *NtSOS1* were quantified using qPCR. We found that the relative expression levels of these genes were significantly up-regulated in all the tobacco plants at 7 and 15 dpt but the increase of each gene in the OE-1 and OE-3 lines was significantly higher than that of the CK and EV at both time points (Figures 7A–F). The expression levels of *NtSOD, NtPOD*, and *NtCAT* in the OE-1 and OE-3 lines were 1.66 and 1.55, 2.60 and 2.19, 1.59 and 1.78 times higher than that of CK at 7 and 15 dpt, respectively. The expression levels of *NtHAK1, NtPMA4*, and *NtSOS1* in the OE-1 and OE-3 lines were 4.04 and 3.72, 3.64 and 3.85, 2.80 and 2.84 times higher than that of CK at both time points, respectively.

## Discussion

To withstand the negative effects caused by salt stress, plants have evolved various biochemical and molecular mechanisms, such as regulating osmotic balance and ions balance to respond to the stress (Munns, 1993; Chen and Polle, 2010; Ruiz-Lozano et al., 2012; Gong et al., 2020). A number of TF genes have been reported as having effect on improving tolerance to salt and other abiotic stresses. Numerous studies have shown that plant NAC family members play a key role in response to abiotic stresses. It has been reported that the over-expression of *ONAC063* (Naoki et al., 2009), *GmNAC20* (Yu et al., 2011), *GmNAC11* (Yu et al., 2011), *GmNAC06* (Li et al., 2020), *VvNAC17* (Ju et al., 2020), *MlNAC12* (Yang et al., 2018), and *StNAC053* (Wang et al., 2021) genes can enhance plant salt tolerance. *L. pumilum* is a kind of perennial herb with strong resistance, which is mainly distributed in most areas of north China. A few *NAC* genes from *L. pumilum* have been studied and found to improve salt tolerance (Cao, 2019). In our pre-screening of the salt-tolerance *NAC* family genes in *L. pumilum, LpNAC17* with highly salt-inducible expression was selected for the present study.

*LpNAC17* is expressed in all tissues under normal growth condition. It showed an up-regulated expression following drought, salt, cold, and ABA treatments, suggesting that *LpNAC17* is involved in the ABA signaling pathway. Leaf wilting was found in all tobacco plants under salt stress, but the transgenic lines overexpressing *LpNAC17* suffered less damage. Photosynthesis is one of the important indicators reflecting the response ability of plant photosynthesis to stress (Liu et al., 2013). Studies have shown that salt stress can affect the activity of Chl enzymes and destroy the chloroplast structure, thereby reducing the Chl content in plant leaves (Zhu and Zhu, 1999; Zhou, 2020). In the present study, the contents of Chl and Pn in the transgenic tobacco overexpressing *LpNAC17* was significantly higher than that of the control plants, indicating that *LpNAC17* gene can enhance photosynthesis of tobacco under salt stress by increasing the Chl content. However, the role of *LpNAC17* in promoting Chl synthesis or protecting Chl from degradation under salt stress is unclear and needs further study. In addition, the Tr, Ci, and Gs of the transgenic lines overexpressing *LpNAC17* decreased more rapidly than that of the control plants under salt stress. Many previous studies have also shown that the reductions in these indicators are often accompanied by the reductions in Pn (Farquhar and Sharkey, 1982; Huo et al., 2020; Jia et al., 2021). However, Delatorre-Herrera et al. (2021) found that the rate of CO_2_ assimilation was less dependent on stomatal conductance in the salt-tolerant ecotype (Amarilla) of quinoa. They showed that there is another diffusion mechanism involved in carbon dioxide assimilation, such as mesophyll conduction (Gm), and found that the salt-tolerant ecotype performed better under salt stress mainly due to higher photochemical efficiency and greater ribulose-1,5-bisphosphate carboxylase/oxygenase (RubisCO) activity in that ecotype (Delatorre-Herrera et al., 2021). These findings suggest that plant species and their tolerance mechanisms vary in their gas exchange properties. Therefore, we speculate that the transgenic lines overexpressing *LpNAC17* may also maintain a higher Pn through other mechanisms, such as higher Chl content and higher photochemical efficiency, and greater RubisCO activity. The change of ABA content in plant leaves is caused by salt stress, and ABA is closely related to stomatal closure. Previous studies have shown that overexpressing apple *MdUGT88F4* gene in transgenic lines resulted in lower ABA content under salt stress than that of wild-type plants, and the transcription level of ABA synthesis gene *MdNCED3* in the leaves of each line was increased by salt treatment (Chen, 2021). In the present study, the *LpNAC17* gene was up-regulated by ABA treatment (Figures 2A–C), indicating that *LpNAC17* may regulate the salt tolerance of the transgenic tobacco plants through the ABA signaling pathway, which needs further examination.

Under salt stress, a large amount of ROS is produced. Excessive ROS could increase membrane lipid peroxidation and cause damage to the cellular membrane (Gill and Tuteja, 2010), resulting in the generation of massive secondary products such as MDA that can indirectly reflect ROS in plants (Moore and Roberts, 1998; Farmer and Mueller, 2013). In this study, we found that the content of MDA in *LpNAC17* transgenic plants was lower than that in the control plants under salt stress, indicating that the cell membrane damage of the transgenic plants under salt stress was lower than that of control plants. In order to reduce the damage of salt stress to plants, plants usually increase the concentration of osmotic substances, such as Pro, to improve plant tolerance. Osmotic adjustment substances play roles in protecting cellular macromolecules, protecting the cell membrane structure to improve stress tolerance (Zhang et al., 2020). It has been reported in previous studies that NAC TFs regulate the content of osmotic regulators in plants in response to salt stress. For example, tobacco plants overexpressing *CsNAC1*/*2*/*3* gene accumulated higher levels of osmotically active substances than the control plants under salt stress (Liu et al., 2022), and transgenic plants overexpressing *VuNAC1*/*2* exhibited higher Pro content and lower MDA content than the control plants under salt stress (Srivastava et al., 2022). In the present study, Pro content in the transgenic plants overexpressing *LpNAC17* is significantly higher than in the controls under salt stress. This result implied that *LpNAC17* can reduce the damage to cell membrane under salt stress to a certain extent, and play a role in maintaining the stability of cell membrane under salt stress. These data suggest that over-expression of *LpNAC17* could positively regulate Pro content to confer a better ability to resist salt stress in the transgenic tobacco.

The antioxidant enzymes play an important role in coping with excess ROS to reduce oxidative stress. To maintain cell homeostasis, the antioxidant system in plants plays a dominant role in enhancing the activities of antioxidant enzymes, including SOD, POD, and CAT, to prevent plants from being damaged by salt stress (Jaleel et al., 2009). Antioxidant enzymes can eliminate the damage of ${\text{O}}_{2}^{-}$ and ROS to cells. Many reports indicate that NAC TFs are actively involved in the regulation of ROS metabolism and induction of important ROS scavenging enzymes (Jia et al., 2019; Figueroa et al., 2021; Jin et al., 2021; Mei et al., 2021). Consistent with these studies, we found that under salt stress, the activities of SOD, CAT, and POD were increased in *LpNAC17* over-expression tobacco while the contents of ${\text{O}}_{2}^{-}$ and H_2_O_2_ and the level of ROS were less than those in the control plants. These results indicated that the improvement of ROS homeostasis is an important reason for *LpNAC17*-mediated salt tolerance.

In order to further verify the function of *LpNAC17* under salt stress, the expression levels of several key genes of plant stress tolerance were detected in transgenic plants overexpressing *LpNAC17* after salt stress. The results showed that under salt stress, the expression of genes related to scavenging ROS (*NtPOD, NtSOD, NtCAT*) were induced, and improving plant salt tolerance could be achieved by scavenging excess ROS in plants (Lai et al., 2020). We found that the expression levels of *NtPOD, NtSOD, NtCAT, NtSOS1, NtHAK1*, and *NtPMA4* were significantly up-regulated in the transgenic tobacco overexpressing *LpNAC17* when compared with the control plants, indicating that *LpNAC17* could improve the salt tolerance of transgenic tobacco by increasing the expression of ROS genes and sodium-potassium ion balance genes. *SOS1* plays a role in the process of Na^+^ efflux from cells, which can protect plants from salt poisoning caused by excessive intracellular Na^+^ (Eduardo, 2000). *PMA4* can regulate cellular K^+^ uptake and balance K^+^ concentration (Moriau et al., 1993). Moreover, *HAK1* can balance intracellular Na^+^/K^+^ and prevent excessive intracellular Na^+^ content from causing toxicity to cells (Qin et al., 2015).

**Figure 8:**
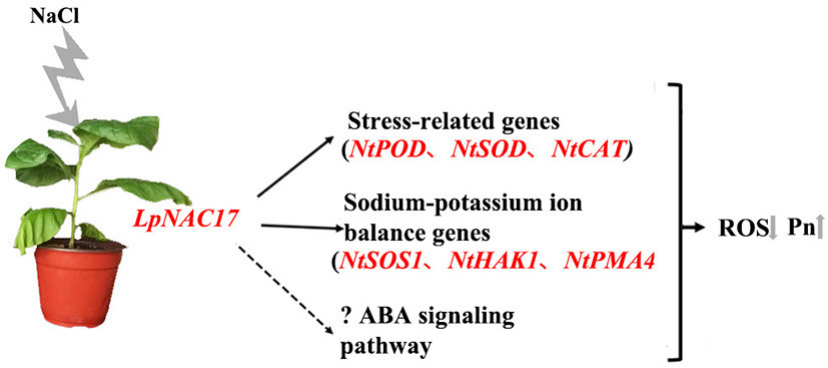
Putative working model of the *LpNAC17* regulatory function in the transgenic tobacco in response to salt stress treatment. Overexpression of *LpNAC17* in tobacco activates the expression of stress-related genes and sodium and potassium ion balance factors, and possibly the ABA signaling pathway, leading to lower accumulation of reactive oxygen species (ROS) and higher Pn under salt stress and thus improved salt tolerance.

We present a working model for the putative regulatory function of *LpNAC17* in responses to salt stress in transgenic tobacco (Figure 8). Overexpression of the TF *LpNAC17* can affect the expression of multiple stress-related genes and indirectly upregulate the activity of ROS scavenging enzymes. Furthermore, increased *LpNAC17* expression may trigger the ABA signaling pathway. Taken together, all these changes resulted in decreased ROS accumulation, enhanced photosynthetic capacity, and increased salt tolerance in transgenic tobacco overexpressing *LpNAC17*.

## Conclusion

In conclusion, a stress-responsive NAC gene *LpNAC17* from *L. pumilum* was identified and its function was characterized. Overexpression of *LpNAC17* enhanced the salinity tolerance of transgenic tobacco at the morphological, physiological and molecular levels. This study provides a functional gene for lily salt tolerance breeding, and lays a foundation for revealing the molecular mechanism of NAC transcription factors under salt stress and for further studying the salt tolerance mechanism in *L. pumilum*.

## Materials and methods

### Gene cloning and sequence analysis

Total RNA was extracted from bulbs of *L. pumilum* and cDNA synthesis was conducted as described in Wang et al. (2020). Gene-specific primers were designed for the *LpNAC17* gene (accession #: MF398208.1) are shown in Supplementary Table S1. PCR amplification was performed with KOD-plus-neo (Toyobo, Japan). The PCR products were digested with Sma*I* and Sal*I* and cloned into the PBI121-GFP empty vector with the *LpNAC17* gene being driven by the full-length 35S promoter, followed by being transformed into *Escherichia coli* DH-5α. After Sanger sequencing confirmation, the destination vector was transformed into *Agrobacterium tumefaciens* EHA105 by the electroporation method.

The amino acid sequence of the *LpNAC17* protein was analyzed using various bioinformatics tools as described in Ma et al. (2017). BLASTp was used to search for homologous sequences in Genbank, and sequences with high sequence homology were selected for sequence alignment. Phylogenetic analysis was constructed by using DNAMAN.

### Tobacco transformation and analysis of transgenic tobacco lines

Tobacco transformation was performed using *Agrobacterium*-mediated transformation of tobacco leaf disks using 50 mg/L Kanamycin for transgenic plant selection (Wang, 2020). Genomic DNA was extracted using the modified CTAB method and PCR was performed as described above. Total RNA extraction and qPCR were conducted as described above. Copy number of the transgene was determined by the segregation ration, and two single-copied homozygous transgenic lines with high transgene expression were selected for subsequent experiments.

### Plant abiotic stress treatment

Twenty-eight-day-old seedlings of *L. pumilum* were pre-cultured with Hoagland's nutrient solution for 1 week and then transferred to Hoagland's nutrient solution containing 200 mM NaCl, 20% PEG or 150 μM ABA. For cold stress, pre-cultured seedlings were transferred to Hoagland's nutrient solution and cultivated in a 2°C artificial climate incubator. Seedlings treated with Hoagland's nutrient solution or 25°C were used as the negative control plants.

Two-week-old transgenic tobacco seedlings overexpressing *LpNAC17* were grown on soil: vermiculite: perlite =3:1:1 culture medium at 26°C with 16 h light/8 h darkness for 3 weeks, followed by the treatment with 100 mL NaCl solution with a concentration of 300 mmol/L once per 2 days. Water was used as the mock treatment. Phenotypic analysis and leaf tissue harvest were conducted at 0, 7 and 15 d of salt stress.

### Real-time RT-PCR analyses

Total RNA was extracted from the roots, bulbs and leaves of *L. pumilum* after each treatment at the time points of 0, 1, 3, 6, 12, 24, or 48 hpt using TRIzol. Total RNA was also extracted from leaves of transgenic tobacco under 300 mM NaCl stress on 0, 7, and 15 dpt. cDNA synthesis was conducted using the ReverTra Ace® qPCR RT Kit (Toyobo, Japan). qPCR was conducted using the 2 × Fast qPCR Master Mixture (CWBIO, China) and 3 biological replicates as described in Zhao et al. (2021). qPCR was performed with the following cycling conditions: 94°C for 2 min followed by 35 cycles at 94°C for 15 s, 60°C for 15 s, and 72°C for 10s. Each 20 μL of reaction mix included 10 μL of 2 × Fast qPCR Master Mixture, 0.4 μL of cDNA, 0.4 μL of Primer-F, 0.4 μL of Primer-R, 8.8 μL of dd H_2_O. The *LilyActin* gene (accession #: JX826390) and the tobacco *NtActin* gene (accession #: no.U60495) were used as the internal control gene for and tobacco, respectively. The relative expression of genes was analyzed using the 2^−ΔΔCt^ method.

### Assays of photosynthetic parameters in tobacco under salt stress

Li-6400 portable photosynthetic instrument (LI-COR, USA) was used to measure the photosynthetic parameters of tobacco plant leaves from 9:00 a.m. to 10:00 a.m. The photosynthetic parameters included leaf net photosynthetic rate (Pn), stomatal conductance (Gs), intercellular CO_2_ concentration (Ci), and transpiration rate (Tr). Before measurement, the photosynthetic instrument was adjusted and preheated. Three plants (replicates) were randomly selected from each line, and the 3rd to 4th leaf were selected from each plant. After the readings stabilized, the data were recorded. Each leaf blade was recorded for 5 times, and the average value was used for each replicate.

### Determination of physiological indexes of transgenic tobacco under salt stress

Malondialdehyde (MDA) accumulation was measured using the thiobarbituric acid-based method (Hodges et al., 1999). The proline (Pro) content was measured using the acid ninhydrin method (Bates et al., 1973). The chlorophyll (Chl) content was determined using the acetone extraction method (Zou et al., 2000; Liu and Li, 2016b,c). The superoxide dismutase (SOD) activity was measured using the nitro blue tetrazolium colorimetric method (Liu and Li, 2016a). The peroxidase (POD) activity was measured using the guaiacol colorimetric method (Zou et al., 2000; Liu and Li, 2016b,c). The catalase (CAT) activity was measured using the ammonium molybdate colorimetric method (Zou et al., 2000; Liu and Li, 2016b,c). The superoxide anion (${\text{O}}_{2}^{-}$) was determined by hydroxylamine oxidation method (Huang et al., 2010). The hydrogen peroxide (H_2_O_2_) content was determined using hydrogen peroxide (H_2_O_2_) content kit (Greis Biotechnology, China). The levels of ${\text{O}}_{2}^{-}$ and H_2_O_2_ were detected with p-Nitro-Blue tetrazolium chloride (NBT) (Liu et al., 2008) and 3,3′- diaminobenzidine tetrahydrochloride (DAB) (Mason et al., 2016) staining, respectively. For the determination of antioxidant enzymes (SOD, POD, and CAT), pre-weighed tobacco leaves (0.5 g) were placed in a pre-cooled mortar, and 5 mL of pre-cooled 50 mmol·L^−1^ (pH 7.8) phosphate buffer (added in several times) was added. The leaves were ground into a homogenate under an ice bath, centrifuged at 3,000 rpm for 10 min at 4°C, and the supernatant was used for the determination of antioxidant enzymes. All these experiments were performed in triplicate.

## Data availability statement

The data presented in the study are deposited in the NCBI repository, accession number: MF398208.

## Author contributions

YpingW, YC, and YZ conceived the project and wrote the manuscript. YpingW, YC, BL, YingW, SS, JW, MT, and HY conducted the experiment, collected the data, and helped with manuscript preparation. YingW, YC, and YZ analyzed the data. All the authors approved the manuscript.

## Funding

This research was funded by the Fundamental Research Funds for Central Universities (2572019BK04) and the Heilongjiang Province Natural Science Foundation (LH2019C004).

## Conflict of interest

The authors declare that the research was conducted in the absence of any commercial or financial relationships that could be construed as a potential conflict of interest.

## Publisher's note

All claims expressed in this article are solely those of the authors and do not necessarily represent those of their affiliated organizations, or those of the publisher, the editors and the reviewers. Any product that may be evaluated in this article, or claim that may be made by its manufacturer, is not guaranteed or endorsed by the publisher.
