# Supplementary material for: Lilium pumilum stress-responsive NAC transcription factor LpNAC17 enhances salt stress tolerance in tobacco
Source: Front Plant Sci. 2022 Sep 2;13:993841. doi: 10.3389/fpls.2022.993841 (PMC9478543; doi:10.3389/fpls.2022.993841)
Supplement: Supplementary file 1 [file Table_1.DOCX]

Supplementary Material

**Table S1.** Primer sequence and application

| **Name** | **Forward / Reverse primer sequence（5’-3’）** | **Temperature/℃** | **Application** |
| --- | --- | --- | --- |
| *LpNAC17* | F:GAGGGCTGTGATTCAAAGAG | 53.5℃ | PCR amplification reaction |
| *LpNAC17* | R:GTCCTATTGGAGACGGTAAC |  |  |
| *LilyActin* | F:GACGCTTCATCTCGTCC | 60℃ | Fluorescence quantitative reference genes |
| *LilyActin* | R:CCACAGGTTGCGTTAG |  |  |
| *LpNAC17* | F:GAAGGCGCTGGTGTTCTATG | 54.5℃ | Fluorescence quantitative PCR reaction |
| *LpNAC17* | R:AACCAAACTATGCCGCGTAC |  |  |
| *LpNAC17*-SmaI | F: TCTAGAATGGGCGGTCCAGATC | 60℃ | PCR amplification reaction |
| *LpNAC17*-SalI | R:GTCGACAACCGGCCCCACAATT |  |  |
| pBI121-GFP | F：TCATTTCATTTGGAGAGAACAC | 52℃ | Bacterial liquid PCR amplification reaction |
| pBI121-GFP | R：TTGCCAAATGTTTGAACGATC |  |  |
| *NtHAK1* | F：ATCCACACCGAGCTTGTTTCAGGA | 60℃ | Fluorescence quantitative PCR reaction |
| *NtHAK1* | R：TGGGTCCAATTCTTCCCACCAAGA |  |  |
| *NtSOS1* | F：GCGTGCTTATTTCCACCTTTTG | 60℃ | Fluorescence quantitative PCR reaction |
| *NtSOS1* | R：TTTGATGACGGCTCCCCAGT |  |  |
| *NtPMA4* | F：TTTCCCGAGCACAAGTATGA | 60℃ | Fluorescence quantitative PCR reaction |
| *NtPMA4* | R：GGTAACCTCCAAGAACAACAC |  |  |
| *NtSOD* | F：CTCCTACCGTCGCCAAAT | 60℃ | Fluorescence quantitative PCR reaction |
| *NtSOD* | R：GCCCAACCAAGAGAACCC |  |  |
| *NtCAT* | F：AGGTACCGCTCATTCACACC | 60℃ | Fluorescence quantitative PCR reaction |
| *NtCAT* | R：AAGCAAGCTTTTGACCCAGA |  |  |
| *NtPOD* | F：CCTCAGCTTCAAGCATTATGTCCA | 60℃ | Fluorescence quantitative PCR reaction |
| *NtPOD* | R：ACCTTTGTAGAAGCATCGGTCCAC |  |  |
| *NtActin* | F：CGGAATCCACGAGACTAACATACAAC | 60℃ | Fluorescence quantitative PCR reaction |
| *NtActin* | R：GGTGCTGAGGGAAGCCAAGATA |  |  |
